# Neutralizing antibodies and fatigue as predictors of low response to interferon-beta treatment in patients with multiple sclerosis

**DOI:** 10.1186/s12883-014-0215-y

**Published:** 2014-11-30

**Authors:** Philippe Manceau, Clotilde Latarche, Sophie Pittion, Gilles Edan, Jérôme de Sèze, Catherine Massart, Marc Debouverie

**Affiliations:** Department of Neurology, Central Hospital, University Hospital, 54000 Nancy, France; Department of Clinical Epidemiology and Evaluation, Marin Hospital, INSERM CIC-EC, Center of Clinical Epidemiology, 54000 Nancy, France; Department of Neurology, Pontchaillou Hospital, University Hospital, 35000 Rennes, France; INSERM 0203, Centre d’Investigation Clinique, Pontchaillou Hospital, 35000 Rennes, France; Department of Neurology, Hautepierre Hospital, University Hospital, 67000 Strasbourg, France; Department of Hormonology, Pontchaillou Hospital, University Hospital, 35000 Rennes, France; School of Public Health, Faculté de Médecine, Nancy Université, EA 4003, Avenue de la Forêt de Haye, 54500 Vandoeuvre-lès-Nancy, France

**Keywords:** Multiple sclerosis, Neutralizing antibodies, Fatigue, Interferon-beta, Response to treatment

## Abstract

**Background:**

The clinical impact of neutralizing antibodies against interferon-beta (NAb) is controversial. Their presence can lead to a decrease in interferon-beta (IFNβ) efficacy. Fatigue reported in patients with multiple sclerosis (MS) may be associated with an unfavorable clinical course. We conducted a prospective multicentre study to assess the association between response to IFNβ, NAb and fatigue.

**Methods:**

Patients with relapsing-remitting MS on IFNβ treatment were included. During the second year of treatment, the patients were analyzed for NAb status and non-response criteria to IFNβ (number of relapses ≥1 during the follow-up period, increase in the Expanded Disability Status Scale ≥0.5). The score on the Modified Fatigue Impact Scale (MFIS pathological if score ≥35) was noted for each patient.

**Results:**

Of the 176 patients included: 22.3% were NAb positive, 54.5% presented non-response criteria to IFNβ, and 57.4% had a pathological MFIS score. Fatigue was increased in NAb + patients (p = 0.0014) and they were more likely to present non-response criteria to IFNβ (p = 0.041) than NAb- patients. Multivariate logistic regression analysis showed that the presence of NAb was related to fatigue (p = 0.0032) and denoted disease activity in these patients (p = 0.026).

**Conclusions:**

This study demonstrates the impact of NAb on the non-clinical response to IFNβ. Fatigue assessment is an indicator of IFNβ responsiveness and a predictive biomarker of deterioration on patient’s neurological status.

## Background

Immunomodulatory treatment with interferon-beta (IFNβ) is a first-line treatment for patients with relapsing-remitting multiple sclerosis (MS). As with any therapy derived from human recombinant proteins, this treatment has immunogenic properties [1]. Neutralizing antibodies (NAb) against IFNβ develop in 2% to 46% of treated patients [2–14]. This large variability in observed NAb prevalence can be explained by the more immunogenic character of IFNβ-1b compared with IFNβ-1a [13], the increased prevalence of NAb with multi-weekly injections [9], the more immunogenic nature of subcutaneous administration as opposed to intramuscular administration [8,13,14], the dose treatment [7,11] and the different follow-up durations. Moreover, a positive NAb status may be reversible over time [12,13,15]. The origin of the reversibility of NAb status is unknown but the hypothesis of a re-establishment of immune tolerance after a breakdown period with IFNβ-treatment is possible [16]. While it is recognized that NAb has a negative impact on magnetic resonance imaging (MRI), the effect of NAb on clinical outcome remains a subject of debate to this day. Indeed, some studies have found conflicting results regarding the impact of NAb on the clinical response to IFNβ [2,4,5,8–12,14,17,18]. The variability of the results about the impact of NAb may depend on the statistical approaches used in these studies, which should consider that many of the NAb positive patients revert to a NAb negative status over time [19]. Another factor, that can impact on NAb status is the neutralizing assay used in these different studies [19]. The interpretation of the NAb status is consequently difficult for the clinician to analyze. This has given rise to recommendations aimed at facilitating the decision as to whether to test for NAb or not [19].

Fatigue is a symptom reported by 53-92% of patients with MS and is one of its most disabling symptoms [20–23]. Direct involvement of immunological factors has been suggested as a pathophysiological mechanism responsible for fatigue during MS [24,25]. Moreover, the intensity of fatigue (physical and psychosocial fatigue), was statistically correlated with the EDSS (Expanded Disability Status Scale) and physical fatigue was a prognostic marker of a worsening of the disability status after a follow-up period of three years [26,27].

We hypothesized that fatigue could be predictive of non-response to treatment with IFNβ. Accordingly, we studied the association between response to IFNβ, fatigue and the presence of NAb.

## Methods

### Inclusion criteria

To be included patients had to be ≥18 years, IFNβ naive, with an EDSS ≤5.0, and fulfilling the clinical criteria for treatment with IFNβ, i.e., patients with a clinically isolated syndrome (CIS) with an active inflammatory process severe enough to need intravenous corticosteroids, if alternative diagnoses had been excluded, and if these patients were considered at high risk of developing clinically definite MS, or patients with relapsing-remitting MS with at least two relapses within the last two years [28].

### Study design

This was a prospective, multicentre study, in the neurological department of three hospitals in France: Strasbourg, Rennes and Nancy. Patients fulfilling the inclusion criteria underwent two specific consultations. The initial consultation, called the “inclusion consultation”, was performed when IFNβ was initiated. During this inclusion consultation, patient consent was obtained and the pre-IFNβ EDSS was assessed. The choice of the IFNβ was at the discretion of the clinician: intramuscular IFNβ-1a (Avonex®, Biogen Idec, Cambridge, MA, USA), subcutaneous IFNβ-1a (22 or 44 μg REBIF®, Merck Serono, Geneva, Switzerland), subcutaneous IFNβ-1b (BETAFERON®, Bayer Schering, Berlin, Germany; EXTAVIA®, Novartis, Dorval, Canada). A second consultation, called the “follow-up consultation”, was used to assess the clinical response to IFNβ, the fatigue experienced, the severity of the flu-like syndrome and the patient’s mood. This “follow-up consultation” happened during the second year of treatment. This time for this consultation was chosen to systematically test patients for NAb, as recommended by the experts panel recommendations [19]. During this “follow-up” consultation, patients and physicians were not aware of the result of NAb status. A written informed consent for participation in the study was obtained from all patients. Because the protocol of this study did not modify the classical clinical practice, it was not necessary, in accordance with the French law at the beginning of the study, to require the approval of a specific medical ethic committee. Nevertheless, this study was approved by the Neurology Department of Nancy University Hospital and was conducted according to the criteria of the Helsinki Declaration. Moreover, the use of the database was approved by the CNIL (Commission Nationale de l’Informatique et des Libertés – National Commission on Information Technology and Liberties) and the CCTIRS (Comité Consultatif sur le Traitement de l’Information en matière de Recherche dans le domaine de la Santé – Consultative Committee on Clinical Research Data Management).

### Evaluation of response to IFNβ treatment

The response to IFNβ was assessed at a clinical level only. Non-response to IFNβ between the inclusion and follow-up consultations was defined as the occurrence of at least one MS relapse and/or an increase in EDSS ≥0.5, confirmed after six months. A MS relapse was defined as the occurrence, the recurrence or the worsening for more than 24 hours of neurological symptoms and usually ending in a partial or complete remission [29,30]. A patient was considered to be an IFNβ responder if there was no relapse or worsening of EDSS during follow-up period.

### Evaluation of fatigue

Fatigue was assessed during the follow-up consultation with the French version of the MFIS [23,31]. The MFIS is a self-administered questionnaire, validated in French, which explores fatigue experienced during the previous four weeks through a set of 21 items. Each item is marked from zero (“never”) to four (“almost always”). The final score thus ranges from zero to 84. In our study, a patient was considered tired if his MFIS score was ≥35.

### Evaluation of the severity of the flu-like syndrome

Under IFNβ, fatigue may be secondary to the flu-like syndrome. Consequently, the flu-like syndrome was also evaluated in this study, using items 13 to 16 from the MSTCQ (Multiple Sclerosis Treatment Concern Questionnaire) [32]. The MSTCQ is a self-administered questionnaire assessing the overall tolerability of IFNβ. Items 13–16 assess more specifically the flu-like syndrome. Each of these four items is marked from zero to five. The final score thus ranges from zero to 20.

### Evaluation of mood

As a depressive syndrome can be correlated with fatigue during MS, we also assessed mood [26]. For this we used the MADRS (Montgomery and Asberg Depression Rating Scale) which is a hetero-administered questionnaire consisting of 10 items, marked from zero to six and validates in French [33,34]. The final score thus ranges from zero to 60.

### Testing for neutralizing antibodies against IFNβ

NAb testing was systematically carried out by the hormonology department of the University Hospital of Rennes using a method called “Luciferase Reporter Gene Assay” [35]. NAb titers were calculated using the Kawade-Grossberg formula in TRU (Ten fold Reduction neutralizing Unit)/mL [36]. A titer just under 20 TRU/mL was considered negative; between 20 and 100 TRU/mL and 20 to 400 TRU/mL weakly to moderately positive for IFNβ-1a and IFNβ-1b respectively; and over 100 TRU/mL and 400 TRU/mL highly positive for IFNβ-1a and IFNβ-1b respectively. There was no other NAb testing to confirm NAb positive status.

### Statistical analyses

All statistical analyses were performed using the SAS 9.2® software. All figures were created using GraphPad Prism 5®. Bivariate analyses were expressed as mean difference +/− SEM (Standard Error Mean). The quantitative variables were compared by Student *t*-test or Man-Whitney test and qualitative variables by chi-square test or Fisher’s exact test. The correlation between variables was determined by calculating the Spearman coefficient. The multivariate analysis, to eliminate confounding factors, used logistic regression models. This model was preferred to a survival model because all data were available and collected on the anniversary date of 2 years precisely. A p < 0.05 was considered statistically significant.

## Results

### Included patient characteristics

In all 176 patients fulfilled the inclusion criteria and participated in this study: 131 (74.4%) were females, 45 (25.6%) were males giving a sex ratio of 2.91. The average age at MS diagnosis was 34 +/− 0.8 years and at study inclusion 36 +/− 0.8 years. The mean duration of MS course before inclusion was 5.3 +/− 0.4 years. The mean EDSS at introduction of IFNβ was 1.7 +/− 0.1 and the mean number of relapses before treatment 2.4 +/− 0.1. At inclusion consultation, 51 patients were CIS with only one MS relapse. The median time from the inclusion consultation to the follow-up consultation was 411 days. Twenty-eight patients (16.1%) received intramuscular IFNβ-1a, 47 (27%) subcutaneous IFNβ-1b and 99 (56.9%) subcutaneous IFNβ-1a. Thirty-nine patients (22.3%) had developed a positive NAb status (NAb+) in the second year of treatment. Ninety-six patients (54.5%) were classified as non-responders to IFNβ between the two consultations (relapse ≥1 or ΔEDSS ≥0.5) and 101 patients (57.4%) were tired at the follow-up consultation.

### NAb + patients characteristics

The 39 NAb + patients were compared with the 136 patients who remained NAb- during follow-up (Table 1). Both groups of patients were similar in terms of age at MS diagnosis (NAb-: 33.8 +/− 0.9 years vs. NAb+: 35.2 +/− 1.5 years, p = 0.48, Student *t*-test), age at treatment initiation (35.5 +/− 0.9 years vs. 37.3 +/− 1.6 years, p = 0.32, Student *t*-test), duration of disease progression (5.3 +/− 0.5 years vs. 4.9 +/− 0.9 years, p = 0.66, Student *t*-test), EDSS at inclusion consultation (1.7 +/− 0.1 vs. 1.8 +/− 0.2, p = 0.66, Student *t*-test) and number of relapses before inclusion consultation (2.4 +/− 0.1 vs. 2.4 +/− 0.2, p = 0.98, Student *t*-test). There was also no statistically significant difference between the two groups regarding the IFNβ type (intramuscular IFNβ-1a: 25 (18.5%) vs. 3 (7.9%) patients; subcutaneous IFNβ-1a: 77 (57%) vs. 22 (57.9%) patients; subcutaneous IFNβ-1b SC: 33 (24.4%) vs. 13 (34.2%) patients, p = 0.21, Fisher’s exact test). In contrast, there was a statistically significant difference in terms of sex with an F/M sex ratio of 2.32 in the NAb- group vs. 8.75 in the NAb + group (p = 0.012, Fisher’s exact test).

**Table 1:** **Comparison of the different variables between NAb- and NAb + patients**

	**NAb- (n** **=** **136)**	**NAb + (n** **=** **39)**	**p**
**Female sex**	95 (69.9%)	35 (89.7%)	**0.012**
Mean age at MS diagnosis (years +/− SEM)	33.8 +/− 0.9	35.2 +/− 1.5	0.48
Mean age at treatment initiation (years +/− SEM)	35.5 +/− 0.9	37.3 +/− 1.6	0.32
Mean duration of MS course (years +/− SEM)	5.3 +/− 0.5	4.9 +/− 0.9	0.66
Mean EDSS at inclusion consultation (+/− SEM)	1.7 +/− 0.1	1.8 +/− 0.2	0.66
**Mean** ΔEDSS* **(+/− SEM)**	+0.1 +/− 0.1	+0.4 +/− 0.1	**0.035**
Mean number of relapse before inclusion consultation (+/− SEM)	2.4 +/− 0.1	2.4 +/− 0.2	0.98
**Mean number of relapse between inclusion and follow-up consultations (+/− SEM)**	0.6 +/− 0.1	1.0 +/− 0.2	**0.016**
**Number of non-responder patients**	69 (50.7%)	27 (69.2%)	**0.041**
**Mean MFIS score (+/− SEM)**	34.2 +/− 1.7	47.3 +/− 2.9	**0.0002**
**Number of fatigue patients**	69 (50.7%)	31 (79.5%)	**0.0014**
Mean MADRS score (+/− SEM)	3.6 +/− 0.4	4.8 +/− 0.7	0.14
Mean MSTCQ score (+/− SEM)	7.2 +/− 0.4	5.9 +/− 0.8	0.086
IFNβ type:			
- Intramuscular IFNβ-1a	25 (18.5%)	3 (7.9%)	0.21
- Subcutaneous IFNβ-1a	77 (57%)	22 (57.9%)	
- Subcutaneous IFNβ-1b	33 (24.4%)	13 (34.2%)	

*ΔEDSS = EDSS at the follow-up consultation – EDSS at the inclusion consultation.

### Relationship between NAb titer and clinical response to IFNβ

**Figure 1:**
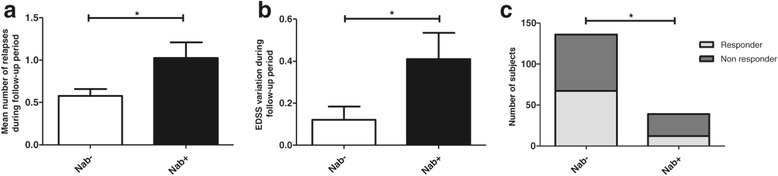
**Comparison of NAb- and NAb + groups in terms of response to IFNβ treatment during the follow-up period. a**. Mean number of relapse during follow-up period between NAb- and NAb + groups. **b**. EDSS variation during follow-up period between NAb- and NAb + groups. **c**. Number of responder and non responder in NAb- and NAb + groups. *p <0.05.

We compared the NAb- group with the NAb + group and studied the impact of NAb on the response to IFNβ. On bivariate analysis, we found a statistically significant difference during the follow-up period in terms of number of relapses (NAb-: 0.6 +/− 0.1 vs. NAb + 1.0 +/− 0.2, p = 0.016, Student *t*-test) and EDSS variation (ΔEDSS = 0.1 +/− 0.1 vs. 0.4 +/− 0.1, p = 0.035, Student *t*-test) (Figure 1a and b). Thus, the ratio Responder/Non-Responder to IFNβ is statistically different between the two groups (Responder/Non-Responder: 67/69 (49.3%/50.7%) vs. 12/27 (30.8%/69.2%), p = 0.041, Chi-square test) (Figure 1c).

Multivariate analysis demonstrated that non-response to IFNβ is the only factor explaining a NAb + status (OR = 2.4, 95% CI: 1.1-5.3; p = 0.026).

### NAb impact on the different scales

**Figure 2:**
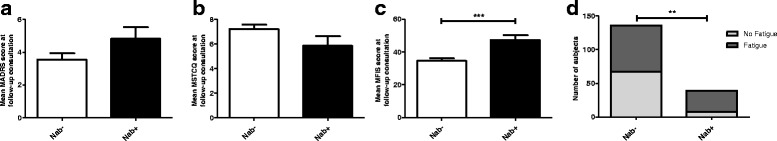
**Comparison between NAb- and NAb + groups on the different scales at the time of the follow-up consultation. a**. Mean MADRS score at follow-up consultation between NAb- and NAb + groups. **b**. Mean MSTCQ score at follow-up consultation between NAb- and NAb + groups. **c**. Mean MFIS score at follow-up consultation between NAb- and NAb + groups. **d**. Number of fatigue and no fatigue in NAb- and NAb + groups. **p <0.01, ***p <0.001.

Bivariate analysis showed a trend for NAb + patients to have a higher MADRS score (4.8 +/− 0.7) than NAb- patients (3.6 +/− 0.4; p = 0.14; Student *t*-test) (Figure 2a). NAb + patients tend to be less affected than NAb- patients by the flu-like syndrome as assessed by items 13 to 16 of the MSTCQ (5.9 +/− 0.8 vs. 7.2 +/− 0.4; p = 0.086; Student *t*-test) (Figure 2b). In terms of fatigue on the MFIS score, there was a significant difference between the NAb- group (34.2 +/− 1.7) and the NAb + group (47.3 +/− 2.9; p = 0.0002; Student *t*-test) (Figure 2c). Also, the ratio No fatigue patients/Fatigue patients was significantly different between the NAb- group (67/69) and the NAb + group (8/31; p = 0.0014, Chi-square test) (Figure 2d).

### Relationship between response to IFNβ and fatigue

**Figure 3:**
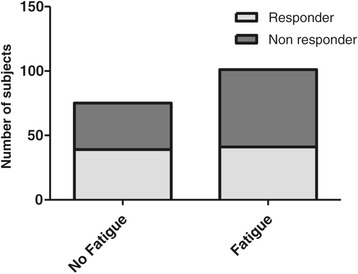
**Comparison of No fatigue and Fatigue groups in terms of response to IFNβ treatment.**

Bivariate analysis did not reveal any significant differences but only a clear tendency for fatigue patients to be non-responders to IFNβ (Responder/Non-Responder: 41/60 (40.6%/59.4%)) compared with patients without fatigue (39/36 (52.0%/48.0%); p = 0.13; Chi-square test) (Figure 3).

### NAb titer and fatigue intensity

**Figure 4:**
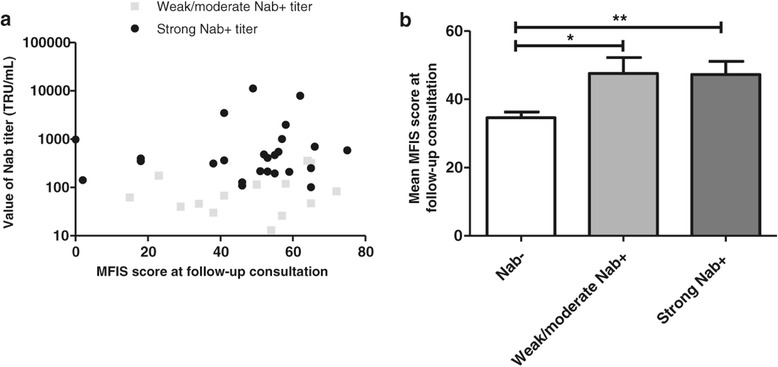
**Influence of NAb titer on MFIS score at follow-up consultation. a**. NAb titer depending on MFIS score. **b**. Comparison of mean MFIS score at follow-up consultation between NAb- patients, weak/moderate NAb + patients and strong NAb + patients. *p <0.05, **p <0.01.

Among the 39 NAb + patients, the mean NAb titer was 878 +/− 346 TRU/mL. Our study did not reveal any correlation between the NAb titer and MFIS score (Spearman coefficient r = 0.14; p = 0.38) (Figure 4a).

Fourteen patients (35.9%) were classified as weakly or moderately positive and 25 patients (64.1%) had a strong positive titer. The mean MFIS score for patients with a low to moderate positive titer was statistically similar to the score for patients with a strong positive titer (respectively 47.5 +/− 4.7 and 47.2 +/− 3.8; p = 0.98; Man-Whitney test) (Figure 4b).

### A positive NAb titer is related to female sex and fatigue and is inversely related to the intensity of flu-like syndrome

Multivariate analysis demonstrated a statistically significant association between NAb + and fatigue (OR = 4.6; 95% CI: 1.7 to 12.8; p = 0.0032). There was also a statistically significant relation between the female sex and NAb + (OR = 3.4; 95% CI: 1.05 to 11.0; p = 0.04). Moreover, the intensity of the flu-like syndrome was inversely correlated to the probability of developing a NAb + titer (OR = 0.88; 95% CI: 0.80-0.97; p = 0.01).

### CIS subgroup of patients

Among the 51 CIS patients at inclusion consultation, 23 (45.1%) converted into a clinically definite MS. These 23 patients were compared to the 28 CIS patients without conversion to a clinically definite MS. There was no statistically significant difference between these two groups in terms of Nab status, EDSS score, fatigue, depression and flu-like syndrome intensity (Table 2).

**Table 2:** **Comparison of the different variables on CIS patients with or without conversion to clinically definite MS at the end of follow-up period**

	**CIS patients with conversion to clinically definite MS (n** **=** **23)**	**CIS patients without conversion to clinically definite MS (n** **=** **28)**	**p**
Female sex	17 (73.9%)	21 (75%)	0.93
Mean age at MS diagnosis (years +/− SEM)	31.8 +/− 2.2	31.0 +/− 2.0	0.79
Mean age at treatment initiation (years +/− SEM)	33.4 +/− 2.5	32.1 +/− 2.0	0.69
Mean EDSS at inclusion consultation (+/− SEM)	1.2 +/− 0.2	1.3 +/− 0.2	0.77
Mean ΔEDSS* (+/− SEM)	+0.2 +/− 0.04	0 +/− 0.02	0.70
Mean MFIS score (+/− SEM)	36.0 +/− 4.5	35.8 +/− 3.3	0.98
Number of fatigue patients	13 (56.5%)	16 (57.1%)	0.96
Mean MADRS score (+/− SEM)	3.7 +/− 0.8	3.2 +/− 0.5	0.63
Mean MSTCQ score (+/− SEM)	6.3 +/− 0.8	6.3 +/− 0.9	0.99
Number of NAb + patients	5 (21.7%)	7 (25.0%)	0.78
IFNβ type:			
- Intramuscular IFNβ-1a	2 (8.7%)	3 (10.7%)	0.43
- Subcutaneous IFNβ-1a	16 (69.6%)	15 (53.6%)	
- Subcutaneous IFNβ-1b	5 (21.7%)	10 (35.7%)	

*ΔEDSS = EDSS at the follow-up consultation – EDSS at the inclusion consultation.

## Discussion

Our study demonstrates the impact of NAb on the non-clinical response to IFNβ in relapsing-remitting MS patients. The clinical impact is characterized both by the existence of relapses during follow-up and by the worsening of EDSS at the end of the follow-up period. Our hypothesis that fatigue could be predictive of a deterioration of the patient’s neurological status can be tentatively confirmed. Nevertheless, this hypothesis is supported by the demonstration of a link between the presence of NAb and fatigue, NAb being involved in non-response to IFNβ. In addition, our study suggests that women are at greater risk of developing NAb. We show that the presence of the flu-like syndrome during follow-up is predictive for the absence of NAb.

The non-response to IFNβ in these patients reflects both the number of relapses during follow-up and changes in EDSS. To our knowledge, only one study to date has demonstrated the negative role of these two clinical parameters but this study only included 78 patients [10]. Other studies have found divergent results and are summarized in Table 3 [2,4,5,8–12,14,17,18].

**Table 3:** **Summary of the literature on clinical impact of NAb in terms of annualized relapse rate, EDSS and MRI activity**

**Study**	**Annualized relapse rate (Number of relapses/year)**			**EDSS score impact**	**MRI impact**	**Study duration**
	**NAb+**	**NAb-**	**p**			
**IFN** **β** **Multiple Sclerosis Study Group, University of British Columbia MS/MRI Analysis Group, Neurology 1996** **[** 2 **]**	1.16	0.50	< 0.05	Positive impact	Yes	3 years
**Ruddick et al., Neurology 1998** **[** 4 **]**	0.50	0.65	NS	No impact	Yes	2 years
**PRISMS Study Group, Lancet 1998** **[** 5 **]**	1.75	1.74	_	_	_	2 years
**Panitch et al., Neurology 2002** **[** 8 **]**	_	_	NS	_	Yes	48 weeks
**Sorensen et al., Lancet 2003** **[** 9 **]**	0.64	0.43	< 0.03	No impact	_	_
**Malucchi et al., Neurology 2004** **[** 10 **]**	0.85	0.53	0.039	Negative impact	_	3 years
**Francis et al., Neurology 2005** **[** 17 **]**	0.85	0.52	< 0.001	No impact	Yes	4 years
**Kappos et al., Neurology 2005** **[** 18 **]**	0.97	0.70	0.04	No impact	Yes	4 years
**Freedman et al., Multiple Sclerosis 2005** **[** 11 **]**	_	_	NS	No impact	Yes	3 years
**Hartung et al., Neurology 2011** **[** 12 **]**	_	_	0.39	No impact	Yes	5 years
**Sato et al., Tokohu J Exp Med 2012** **[** 14 **]**	**_**	**_**	0.13	_	Yes	**_**

These differences in results can be explained by methodological differences from one study to another, especially in the duration of the follow-up period. The median follow-up of patients in our study was 411 days which is a short follow-up duration. Thus, it could be envisaged that the clinical impact of NAb on the non-response to IFNβ is expressed mainly at the beginning of treatment and that the negative clinical impact of NAb would tend to decrease over time. This hypothesis is supported by the possibility of a NAb reversibility [12,13,15]. However, we can conclude that the presence of NAb is a marker for therapeutic ineffectiveness to IFNβ at the beginning of treatment.

The various IFNβ available for the clinician have different immunogenicity properties [7–9,11,13,14]. In terms of non-response to IFNβ induced by NAb, our study would point towards the use of the least immunogenic IFNβ, i.e., intramuscular IFNβ-1a. To our knowledge, no study has compared the therapeutic response to the different forms of IFNβ on the annualized relapse rate and EDSS progression, except the work of Etemadifar et al. which was on a small population of 90 patients [37]. In the same vein, our study found that the risk of developing NAb was higher for women than for men. This finding could suggest a better clinical response to IFNβ in male patients. Few studies have focused on the clinical response to IFNβ according to gender. One study, by Rudick et al., found no difference in intramuscular IFNβ-1a efficacy between men and women but this result may be biased due to the low prevalence of NAb in the study as intramuscular IFNβ-1a is the least immunogenic IFNβ [38].

Fatigue could be the sign of a negative clinical course of MS and may even be the only manifestation of an authentic relapse [39]. In our work, we found a clear trend for non-responder patients to IFNβ to be more tired than responder patients. It has already been suggested in the past that fatigue is a prognostic marker for an unfavorable neurological outcome [27]. We demonstrate a highly significant relation between fatigue and Nab + status. As Nab + status is related to non-response to IFNβ, we believe that fatigue is a clinical marker for non-response to IFNβ. Thus, it would seem appropriate to propose that tired patients under IFNβ be tested for NAb and, if a NAb + status is detected, to consider an alternative therapy. Thereby, in 2008, Capobianco et al. demonstrated the advantage of a treatment change to glatiramer acetate in NAb + patients with a gain of 20.1 months without relapse in this setting [40]. Our work demonstrates that the intensity of fatigue is not correlated to the value of the NAb titer. Thus, in the hypothesis that fatigue is a reflection of non-response to IFNβ treatment, a high NAb titer would not denote a poorer clinical response than a low or a moderate titer.

Nevertheless, this study demonstrates that fatigue can also occur in patients with NAb negative status. It leads to consider that fatigue during MS is not only related with NAb status. Indeed, there are several dimensions to MS-related fatigue [41,42]. Primary fatigue is directly related to the pathophysiological mechanisms associated with MS, including direct involvement of immunological factors [24,25]. Secondary fatigue is explained by the direct effects of the disease (sleep disorder, reactive depression, analgesic treatment of nociceptive pain, etc.).

Interestingly, the occurrence of the flu-like syndrome under IFNβ seems to be prognostic for NAb- status. Indeed, the intensity of the flu-like syndrome is inversely correlated to the risk of being NAb+. The flu-like syndrome is one of the major causes for patients to stop a long-term IFNβ treatment. Hartung et al. found a similar result in their study: a clear trend in NAb- patients to stop their treatment with IFNβ compared with NAb + patients due to the adverse effects of the treatment which were more prevalent in the former [12]. Therefore, the existence of a flu-like syndrome during the follow-up of patients receiving IFNβ is a good prognostic factor for the absence of NAb and, paradoxically, would suggest that treatment should be maintained. However, this result should be interpreted with caution because the flu-like syndrome in our study was assessed by a subset of the MSTCQ-score. This score, though validated to assess the overall adverse effects of a treatment with IFNβ, has not yet been validated for all the subsets.

Our study has some limitations. Firstly, this work has an open non randomized study design that may influence the results and lead to a selection bias. Furthermore, the work focuses on a relatively small population of 176 patients with a median follow-up just under 14 months. These two parameters give our study a low statistical power to clearly demonstrate a significant relation between fatigue and non-response to IFNβ. The link between fatigue and adverse neurological outcome in the work of Debouverie et al. was demonstrated after a 3-year follow-up [27]. We believe that a longer follow-up on a larger population would allow us to confirm this correlation. In addition, non-response to IFNβ can also be evaluated radiologically and not only clinically. It is possible that some of our patients who were considered IFNβ responders could have a radiological activity of the disease. This might be a bias in our study in the classification of patients. Moreover, it would be interesting to know for NAb + patients the time period spent with NAb- status, that can also impact the interpretation of the results [19].

## Conclusion

Our study establishes the impact of NAb on the non-response to IFNβ in patients with relapsing-remitting MS. Moreover, there is a tripartite relation between response to IFNβ, fatigue and NAb status. NAb positive status is correlated with an increased prevalence of fatigue during follow-up. Although the link between fatigue and non-response to IFNβ cannot be demonstrated directly because of a lack of statistical power, we found a clear trend to suggest a link between these two parameters. Thus, fatigue in patients treated with IFNβ constitutes a good clinical marker for non-response to treatment. Detection of fatigue by a validated scale should lead the clinician to test for NAb status and possibly to propose an alternative therapeutic treatment to IFNβ.

## Abbreviations

CIS: Clinically isolated syndrome
EDSS: Expanded disability status scale
IFNβ: Interferon-beta
MADRS: Montgomery and asberg depression rating scale
MFIS: Modified fatigue impact scale
MS: Multiple sclerosis
MSTCQ: Multiple sclerosis treatment concern questionnaire
MRI: Magnetic resonance imaging
NAb: Neutralizing antibodies against interferon-beta
OD: Odd ratio
SC: Subcutaneous
SEM: Standard error mean
TRU: Ten fold Reduction neutralizing Unit
vs: Versus
